# Using Sparse Parts in Fused Information to Enhance Performance in Latent Low-Rank Representation-Based Fusion of Visible and Infrared Images

**DOI:** 10.3390/s24051514

**Published:** 2024-02-26

**Authors:** Chen-Yu Hao, Yao-Chung Chen, Fang-Shii Ning, Tien-Yin Chou, Mei-Hsin Chen

**Affiliations:** 1 GIS Research Center, Feng Chia University, Taichung 40724, Taiwan; how@gis.tw (C.-Y.H.); jimmy@gis.tw (T.-Y.C.); ivy@gis.tw (M.-H.C.); 2 Department of Land Economics, National Chengchi University, Taipei 11605, Taiwan; fsn@nccu.edu.tw

**Keywords:** Latent Low-Rank Representation (LatLRR), sparse part, Convolutional Neural Network (CNN), VGG19, ResNet50, image fusion

## Abstract

Latent Low-Rank Representation (LatLRR) has emerged as a prominent approach for fusing visible and infrared images. In this approach, images are decomposed into three fundamental components: the base part, salient part, and sparse part. The aim is to blend the base and salient features to reconstruct images accurately. However, existing methods often focus more on combining the base and salient parts, neglecting the importance of the sparse component, whereas we advocate for the comprehensive inclusion of all three parts generated from LatLRR image decomposition into the image fusion process, a novel proposition introduced in this study. Moreover, the effective integration of Convolutional Neural Network (CNN) technology with LatLRR remains challenging, particularly after the inclusion of sparse parts. This study utilizes fusion strategies involving weighted average, summation, VGG19, and ResNet50 in various combinations to analyze the fusion performance following the introduction of sparse parts. The research findings show a significant enhancement in fusion performance achieved through the inclusion of sparse parts in the fusion process. The suggested fusion strategy involves employing deep learning techniques for fusing both base parts and sparse parts while utilizing a summation strategy for the fusion of salient parts. The findings improve the performance of LatLRR-based methods and offer valuable insights for enhancement, leading to advancements in the field of image fusion.

## 1. Introduction

Image fusion, particularly the integration of visible and infrared images, has become an interesting and demanding research area in recent years. Visible images offer rich color and texture information, while infrared images succeed in capturing thermal radiation data in low-light conditions. The fusion of these image modalities yields valuable insights for a wide range of applications, such as intelligent urban surveillance [1], environmental monitoring [2], autonomous vehicles [3], medical diagnostics [4,5], military surveillance [6], and precision weapon targeting. Researchers in this domain have diligently advanced various methods, classifiable into three main categories based on their processing techniques: multi-scale transformation, sparse representation, and deep learning [7,8,9].

Multi-scale transformation is a method that primarily involves the decomposition of the original image into multiple scales, resulting in sub-images at different spatial scales. Common methods for this decomposition include wavelet transforms [10], pyramid transforms [11], contourlet transforms (CT) [12], non-subsampled contourlet transforms (NSCT) [13], fourth-order partial differential equations (FPDEs) [14], anisotropic diffusion [15], and shift-invariant shearlet transforms [16]. Following this decomposition, pixel-level or region-level fusion strategies are applied, which include techniques such as weight allocation and combination methods like maximum, average, and weighted average. Subsequently, the final fused image is reconstructed from the fused sub-images using an inverse multi-scale transformation. This approach is widely used in various applications and research contexts.

The fundamental concept of sparse representation (SR) [17,18,19,20,21] posits that image signals can be represented as a linear combination of a select few atoms drawn from a pre-learned dictionary, with the sparse coefficients capturing the salient characteristics of the source images.

These two categories correspond to conventional approaches for fusing visible and infrared images. In recent years, there has been widespread adoption of deep learning in this field. Typically, these approaches can be further subdivided into four categories depending on the specific methodologies applied:

- Convolutional Neural Network (CNN)-based methods can be categorized into two primary methods. First, CNNs are trained on visible, infrared, and fused images to acquire the requisite weightings for fusion [22,23,24,25,26,27,28,29]. Second, it leverages pre-trained neural network models to only extract features and obtain weight maps from the images, thereby achieving the fusion objective [30,31,32,33];
- Generative Adversarial Network (GAN)-based methods transform the task of integrating visible and infrared images into an adversarial process, characterized by the interplay between a generator and a discriminator. Their objective is to combine visible and infrared images through the generator, at the same time tasking the discriminator with evaluating the sufficiency of visible and infrared information within the fused image [34,35,36,37,38,39,40];
- Encoder-decoder-based networks consist of two main components: an encoder and a decoder. The encoder extracts high-dimensional feature representations from the source images. The decoder’s job is to reconstruct the encoded features, gradually restoring the image’s details and structure, ultimately producing the fused image. Traditional autoencoders typically employ fully connected layers. Convolutional layers and pooling layers have also been utilized, thus improving feature extraction capabilities and robustness [41,42,43,44,45,46];
- Transformer-based methods: the Transformer was originally introduced for natural language processing and has demonstrated significant achievements in this domain [47]. Due to its remarkable long-range modeling capabilities, the Transformer has attracted the attention of researchers in the field of image fusion [48,49,50,51,52,53]. Transformer converters incorporate Multilayer Perceptron (MLP) and Multihead Self-Attention (MSA) blocks. Residual structures and Layer Normalization (LN) are applied before each MSA and MLP layer. The core design of these converters involves the fusion of input vectors with positional embeddings to preserve positional information for each vector.

Latent Low-Rank Representation (LatLRR) has emerged as a recently employed method for image fusion [54,55,56,57,58]. LatLRR decomposes images into three components: base, salient, and sparse parts [59]. A fusion strategy is then applied to merge the extracted features from the base and salient parts. Typically, it is common to use the average of base parts and the summation of salient parts. Lately, the integration of LatLRR with CNN-based methods has been proposed. This integration is aimed at further enhancing the quality and effectiveness of the fusion process, ultimately resulting in improved fused images. Nevertheless, it is noteworthy that existing LatLRR-based methods suffer from certain limitations as indicated in the literature. Firstly, the current approaches mainly concentrate on the base and salient parts, forgetting about the sparse parts. Furthermore, the proficient development of fusion strategies, particularly in the integration of CNN technology, continues to be a crucial element influencing the overall performance of fusion processes.

Taking these issues into account, this study incorporates all three components obtained from LatLRR image decomposition, namely the base, salient, and sparse parts, into the image fusion process. VGG19 and ResNet50 are separately employed as methods to obtain weight maps. The investigation seeks to evaluate the impact of including the sparse parts on fusion performance and identify the most appropriate fusion strategy, effectively leveraging the advantages of hybrid methods.

## 2. Related Work

LatLRR is an image decomposition method initially introduced by Liu et al. [59] in 2011, serving as an enhancement over the Low-Rank Representation (LRR) proposed in 2010 [60]. This development aimed to address LRR’s constraint in extracting local structures from raw data. In 2018, Li et al. [54] utilized LatLRR for the fusion of visible and infrared images. Their methodology involved utilizing a weighted average to combine the base parts, while employing the summation strategy to combine the salient parts, resulting in the creation of the final fused image. Following this, in 2020, Li et al. [56] introduced a multi-level decomposition approach named MDLatLRR for image decomposition. Additionally, they crafted a fusion framework based on MDLatLRR for the fusion of visible and infrared images. The MDLatLRR method facilitates the extraction of multi-level salient features. It leverages the weighted average to obtain the fused base parts and utilizes the nuclear norm to compute the weights for the fusion of salient parts.

After the application of LatLRR in the fusion of visible and infrared images, several studies have proposed fusion methods that combine LatLRR with other feature extraction techniques. The primary concept is to use LatLRR for image decomposition and then employ various techniques to fuse the base parts or the salient parts. In 2021, Huang and colleagues [58] introduced a method that combines LatLRR with Independent Component Analysis (ICA). This method uses ICA to fuse the base parts, while the salient parts are fused using a summation strategy. In 2022, Prema and others [57] proposed a fusion method that combines LatLRR with ResNet. They used ResNet50 to fuse the salient parts, and the base parts were fused using a weighted average strategy. Tao et al. [61] proposed LatLRR-VGG19 which uses VGG19 to fuse the base parts, while the salient parts are fused using a summation strategy. In 2023, Yang and his team [55] presented LatLRR-CNN, where both the base parts and the salient parts were initially fused using CNN, and the final fused image was obtained by summing the two. These studies aim to enhance image fusion performance by leveraging LatLRR and various other feature extraction techniques.

LatLRR has been verified as a robust and efficacious approach for image decomposition, especially in the context of fusing visible and infrared images. The key point of fusion strategies using LatLRR lies in the judicious application of a good weight map extraction methodology and developing a suitable mechanism for the optimal integration of each separated component. It is noteworthy that the extant literature on visible and infrared image fusion methodologies based on LatLRR has tended to overlook sparse parts. This oversight may result in the loss of specific features inherent in the original images during the fusion process. Additionally, the determination of the application of CNN-based weight map extraction methods for specific parts represents a pivotal factor influencing fusion performance. These considerations serve as the primary focus of inquiry in this study.

## 3. Methodology

This study focuses on suggesting a new way to combine images using the LatLRR method. We also want to look at the sparse parts of the images usually seen as noise and removed. We think the sparse parts might have important information. Our main idea is to show that this part should not be ignored. The main goal is to prove this with real evidence. To achieve this, we use the LatLRR fusion method as our base. We choose methods that have performed well in traditional approaches without incorporating sparse parts. We pay close attention to how we design our fusion strategies and carefully analyze the results. By building on what others have done before, we can test and show the differences between our method, which includes the sparse parts, and other methods that do not.

Through this careful analysis, we hope to provide strong evidence that the LatLRR-based image fusion method really does improve how well it works. At the same time, we aim to share useful insights that can help make progress in the field of image fusion.

### 3.1. LatLRR for Image Decomposition

In this study, LatLRR is employed to decompose visible and infrared images into base parts, salient parts, and sparse parts. In reference [59], LatLRR, by solving a nuclear norm minimization problem, can approximate the recovery of hidden data influences. The optimization problem can be expressed as Equation (1):

$$\underset{Z,L,E}{\min}{\left\|Z\right\|}_{*}+{\left\|L\right\|}_{*}+\lambda {\left\|E\right\|}_{1}$$

**Figure 1:**
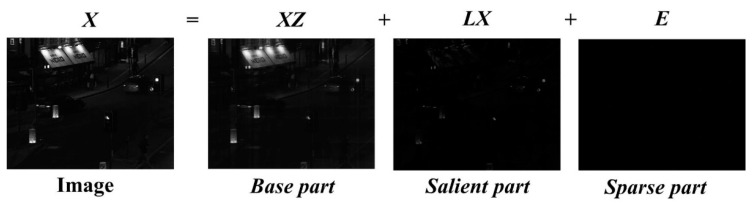
The three parts of a decomposed image using LatLRR.

(1) s.t.,X=XZ+LX+E

where *λ* is the balance coefficient and is greater than 0, ${\left\|\cdot \right\|}_{*}$ denotes the nuclear norm, which is the sum of the singular values of the matrix, and ${\left\|\cdot \right\|}_{1}$ represents the *l*_1_-norm. *X* represents the observed data matrix, *Z* is the low-rank coefficient, *L* is the salient coefficient, and *E* is the sparse spart. Equation (1) could be solved using the inexact Augmented Lagrangian Multiplier (ALM) [59] algorithm. Then, the base part *XZ*, salient part *LX*, and sparse part *E* are derived from Equation (1), as illustrated in Figure 1.

In the context of LatLRR’s image decomposition algorithm, the sparse part is classified as sparse noise. However, theoretically, this part derived from the original image might encapsulate significant image information. Disregarding it in the image fusion process could potentially lead to the forfeiture of valuable image information, consequently inducing distortion in the fused image. Thus, within this study, particular emphasis is placed on integrating the sparse parts meticulously into the image fusion phase to mitigate such potential loss of critical image information.

### 3.2. CNN-Based Pre-Trained Model for Weighted Maps Extraction

As CNNs’ capability in feature extraction has gained widespread acknowledgment, this study adopts a CNN-based pre-trained model to obtain the weighted maps necessary for the image fusion process. The advantage of using this method lies in obviating the need for retraining deep learning models or designing loss functions. This approach facilitates the ease of implementation for the fusion strategy designed in this study, thereby offering convenience for subsequent applications of interest to stakeholders.

Li et al. [31] employed the VGG19 model for extracting multi-layer features of the detailed image parts. Following this, they utilized the *l*_1_ norm and a weighted average strategy to generate multiple candidate options for the fused detailed part. Ultimately, employing a maximum selection strategy, they derived the definitive fused detailed content. This content was then integrated with the fused base parts to reconstruct the final fused image. Continuing the study by Li et al. [6], the reutilization of ResNet50 for extracting deep features from the source images is proposed. Subsequently, normalization of the deep features is conducted utilizing Zero-Phase Component Analysis (ZCA) and the *l*_1_ norm to derive initial weight maps. The final weight maps are acquired through a soft-max operation, jointly applied to the initial weight maps. Ultimately, a fused image is reconstructed employing a weighted average strategy. Inspired by the literature, this study will utilize VGG19 and ResNet50 as neural network models for feature extraction.

### 3.3. The Fusion Strategy

**Figure 2:**
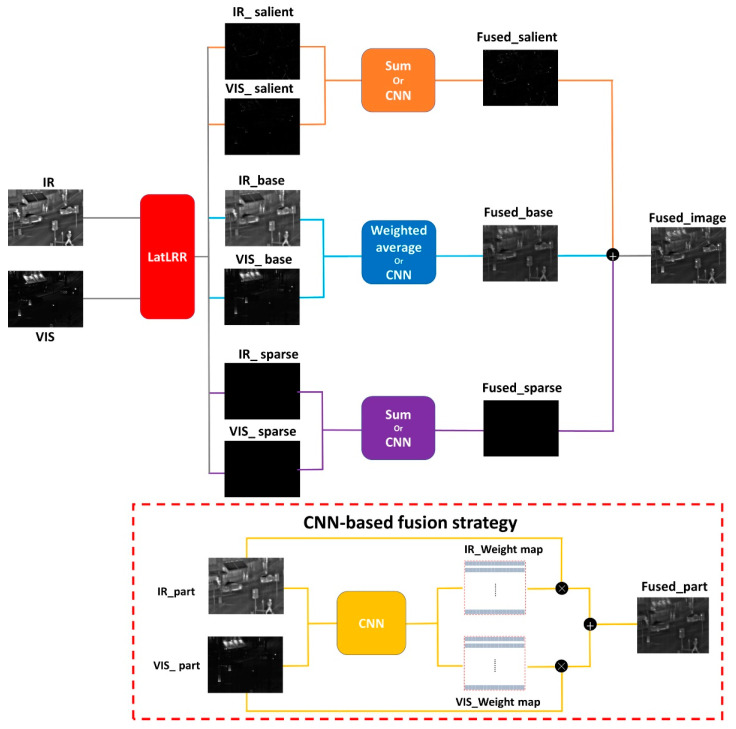
The framework of the proposed method.

The image fusion techniques devised in this study institute comprise four methods: weighted average, summation, and the utilization of VGG19_*l*_1_ norm [31] and ResNet50_ZCA_*l*_1_ norm [30] to derive weight maps. The weighted average method is specifically applied to the base parts, while the summation method is employed for the salient and sparse parts. Additionally, VGG19_*l*_1_ norm and ResNet50_ZCA_*l*_1_ norm are utilized for all three parts. It is important to note that VGG19 and ResNet50 are used independently and not concurrently within the same strategy. The proposed framework is described in Figure 2. The fusion methodologies for weighted average and summation are mathematically expressed in Equations (2) and (3):

(2) $$IM_{weighted\_average}=weight_{IR}\times IR_{part}+weight_{VIS}\times VIS_{part}$$

(3) $$IM_{summation}=IR_{part}+VIS_{part}$$

where $IM_{weighted\_average}$ represents the fused portion obtained through the weighted average method, $IM_{\mathrm{summation}}$ signifies the fused part derived using the summation strategy, $IR_{part}\;$ denotes the decomposed component extracted from the infrared image, while $VIS_{part}$ represents the decomposed segment from the visible image. $weight_{IR}$ corresponds to the weight attributed to $IR_{part}$, and $weight_{WIS}$ pertains to the weight assigned to $VIS_{part}$. In this study, both weights were set to 0.5.

The methodologies referred to as VGG19_*l*_1_ norm and ResNet50_ZCA_*l*_1_ norm employ CNN models for feature map extraction. Following this step, the application of the *l*_1_ norm and a combined ZCA with *l*_1_ norm operation is utilized to reduce feature dimensionality while preserving crucial features, thereby obtaining weighted maps for subsequent fusion. Detailed procedural information regarding these methodologies can be found in the literature [5,6], with the expressions described as VGG19+*l*_1_ norm in Equations (4) and (5).

(4) $$F_{fused\_part}^{i}=\sum_{n=1}^{K}W_{n}^{i}\times I_{n}^{part},\;K=2$$

(5) $$F_{fused\_part}=max\left[F_{fused\_part}^{i}|i\in \left\{1,2,3,4\right\}\right]$$

Here, $F_{fused\_part}^{i}$ represents the multi-layer fused part, $W_{n}^{i}$ denotes the weight maps extracted using VGG19, $I_{n}^{part}$ refers to the decomposed parts from both visible and infrared images, K represents the number of image modalities, while i stands for the number of layers, and $F_{fused\_part}$ symbolizes the ultimate fused part.

ResNet50_ZCA_*l*_1_ norm is represented through Equations (6)–(10).

(6) $$Cov_{i}^{j}=F_{i}^{j}\times {\left(F_{i}^{j}\right)}^{T}$$

(7) $$\left[U,\Sigma ,V\right]=SVD\left(Cov_{i}^{j}\right)\;\;\;\;\;s.t.,\;Cov_{i}^{j}=U\Sigma V^{T}$$

(8) $$F_{i}^{P,j}=s_{i}^{P,j}\times F_{i}^{j}$$

(9) $$s_{i}^{P,j}=U{\left(\Sigma +\epsilon I\right)}^{-0.5}U^{T}$$

(10) $$W_{i}=\frac{\sum_{E=x-t}^{x+t}\sum_{N=y-t}^{y+t}{\left\|F_{i}^{P,1:c}\left(E,N\right)\right\|}_{1}}{\left(2t+1\right)*\left(2t+1\right)}$$

(11) $$F_{fused\_part}=\sum_{i=1}^{2}W_{i}\times I_{i}^{part}$$

In the equation set, $Cov_{i}^{j}$ represents the covariance matrix, where i∈{1,2} denotes the image modality, and j∈{1,2,…C} signifies the channels of deep features. $F_{i}^{j}$ stands for the deep feature maps extracted by ResNet50. U,Σ,V, represent the correlation matrices resulting from singular value decomposition (SVD). $F_{i}^{P,j}$ represents the deep feature maps after undergoing ZCA projection transformation from $F_{i}^{j}$, and $s_{i}^{P,j}$ denotes the ZCA transformation matrix. *I* denotes the identity matrix, while ϵ serves as a small positive value utilized to stabilize matrix inversion. $W_{i}$ signifies the weight maps resulting from the $F_{i}^{P,j}$ after the application of the *l*_1_ norm operation. Here, *t* represents the window parameter used during the *l*_1_ norm operation, set to 2 in this study. $I_{i}^{part}$ refers to the decomposed parts from both visible and infrared images, $F_{fused\_part}$ symbolizes the ultimate fused part.

Finally, the fusion of visible and infrared images is obtained by summing the three merged parts, as illustrated in Equation (12).

(12) $$Image_{VIF}=VIF_{Base\_part}+VIF_{salient\_part}+VIF_{sparse\_part}$$

### 3.4. Image Dataset

**Figure 3:**
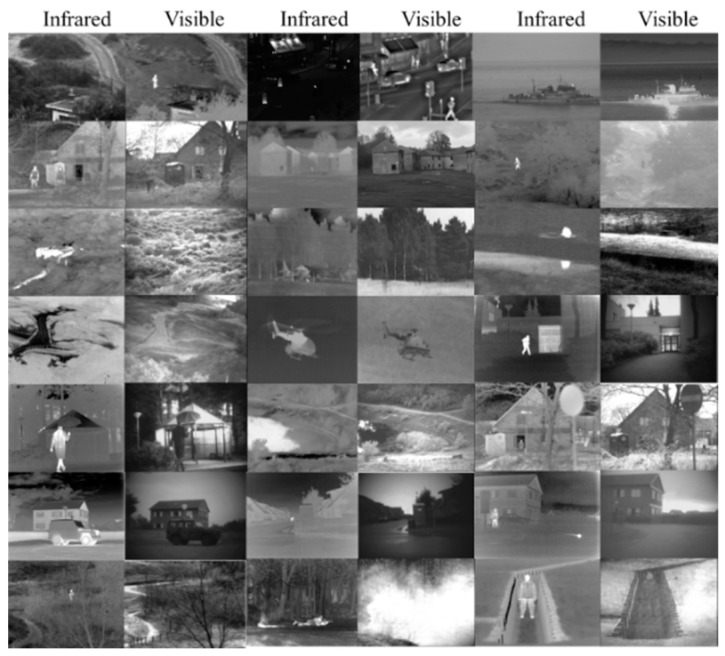
Image pairs of visible images and infrared images (from TNO).

In this study, image pairs consisting of visible images and infrared images were sourced from the TNO image dataset. These image pairs encompass diverse military and surveillance scenarios captured during both day and night periods. They depict various objects and targets, including people, vehicles, ships, and aircraft, against different backgrounds such as rural and urban settings. A total of 21 aligned and processed pairs of visible images and infrared images were selected, as depicted in Figure 3.

## 4. Results

For assessing the quality and performance of the fused images, this study employs a comprehensive evaluation comprising subjective (visual) and objective (quantitative) analyses. Subjective evaluation involves the direct perception and assessment of image quality, considering visual features like clarity, contrast, details, and textures of the fused images based on subjective perception and experience. Objective assessment utilizes quantitative indicators, including Entropy (EN), Mutual Information (MI), Quality Assessment of Fused Band-ratio Images (Qabf), Feature Mutual Information for pixel domain (FMI_pixel), Feature Mutual Information for DCT domain (FMI_dct), Feature Mutual Information for wavelet domain (FMI_w), Normalized Absolute Fused Error (Nabf), Spatial Consistency Deviation (SCD), Structural Similarity Index (SSIM), and Multi-Scale Structural Similarity Index (MS_SSIM). These metrics enable the quantification of properties such as similarity, fidelity, information preservation, and spectral consistency between the fused and original images. In evaluating the performance of the proposed image fusion method, not only were comparisons made among various methods proposed in this study, but also comparisons were conducted with 10 State-of-the-Art methods.

### 4.1. Objective Assessments

This part of the study focuses on measuring the effectiveness of different fusion approaches that include sparse components in the image fusion process. Table 1 and Table 2 present a comparative analysis of quantitative outcomes derived from different fusion techniques, using LatLRR image decomposition, Resnet50_ZCA_*l*_1_ norm, VGG19_*l*_1_ norm, weighted average, and summation. Additionally, these approaches are compared directly with the singular utilization of Resnet50_ZCA_*l*_1_ norm (Resnet50) and VGG19_*l*_1_ norm (VGG19), and the traditional LatLRR (WB + S) methods. The numerical values in these tables signify the absolute comparative performance of each fusion mode across multiple evaluation metrics, where, except for Nabf, higher values indicate superior fusion outcomes across the remaining nine indicators. Consistently, both tables demonstrate an overall enhancement across most evaluation metrics when integrating the sparse parts alongside the base and salient parts in image fusion, compared to strategies that overlook the sparse parts.

By employing a summation strategy for fusing the salient and sparse parts and adopting a CNN-based fusion methodology for the base parts (S_S_ + NB + S), optimal values across metrics such as EN, MI, Qabf, and SCD can be attained. The study introduces two fusion strategies specifically for the sparse parts: summation and CNN-based fusion. While there is no significant discrepancy observed across most indicators between these strategies, the CNN-based fusion strategy demonstrates superior performance in Nabf. Concerning the base parts, CNN-based fusion strategies generally outperform weighted average, albeit with nuanced differences. In terms of the salient parts, a more pronounced disparity exists between CNN-based and summation fusion strategies. The CNN-based fusion manifests superiority in FMI_dct, Nabf, FMI_pixel, and SSIM, whereas the summation strategy excels in EN, MI, Qabf, SCD, and MS_SSIM.

Among the numerous proposed methods, no singular fusion method emerges as universally superior across all 10 assessment metrics. This aligns with the prevalent consensus in this domain that no single fusion method presently exhibits comprehensive advantages. Consequently, the choice of fusion method predominantly hinges upon user-specific requisites. Nevertheless, in an overarching evaluation encompassing multiple metrics, the proposed method employing the CNN-based method for sparse and base parts alongside summation for salient parts (NS_S_ + NB + S) demonstrates robustness, reliability, and comprehensiveness across the metrics.

Table 3 and Table 4 present a comparative analysis of the impact of including or excluding the sparse parts in the image fusion process and employing various fusion strategies. The numerical values in these tables, expressed as percentage improvement rates (%), depict the extent of enhancement observed in different metrics upon integrating the sparse parts into the fusion strategy. The findings reveal that, excluding FMI_pixel and Nabf, most indicators suggest an augmented fusion performance with the inclusion of sparse parts. Notably, FMI_dct, FMI_w, and Nabf exhibit the most pronounced changes compared to other metrics. The utilization of a summation strategy for fusing sparse parts did not improve the performance in the Nabf indicator; instead, it experienced a decline. However, the implementation of a CNN-based method for fusing sparse parts mitigated the earlier limitations and notably amplified the performance in the Nabf indicator. Consequently, an integrated analysis of Table 1, Table 2, Table 3 and Table 4 underscores the efficacy of employing a CNN-based approach for both sparse and base parts, complemented by a summation method for salient parts (NS_S_ + NB + S). This fusion strategy demonstrates relatively superior performance in terms of both image fusion quality and feature similarity. It effectively preserves the inherent characteristics of the original image while minimizing disparities between the fused image and the original, indicating commendable efficacy in information preservation and error reduction.

Table 5 presents a quantitative comparison between the proposed method in this study and 10 existing outstanding methods, encompassing both traditional and deep learning-based approaches. The results show that no single method has absolute superiority. Compared to the 10 State-of-the-Art (SOTA) methods, the proposed method in this study demonstrates better performance in the FMI_dct, FMI_w, Nabf, and SSIM indicators. It also shows impressive performance in the FMI_pixel, SCD, and MS_SSIM indicators, with only slightly poorer performance in EN, MI, and Qabf. These results illustrate that the proposed method not only benefits from incorporating sparse parts but also demonstrates the advantages of combining LatLRR with Resnet50, achieving a complementary effect. It maintains different methods’ performances in individual indicators.

### 4.2. Subjective Assessments

**Figure 4:**
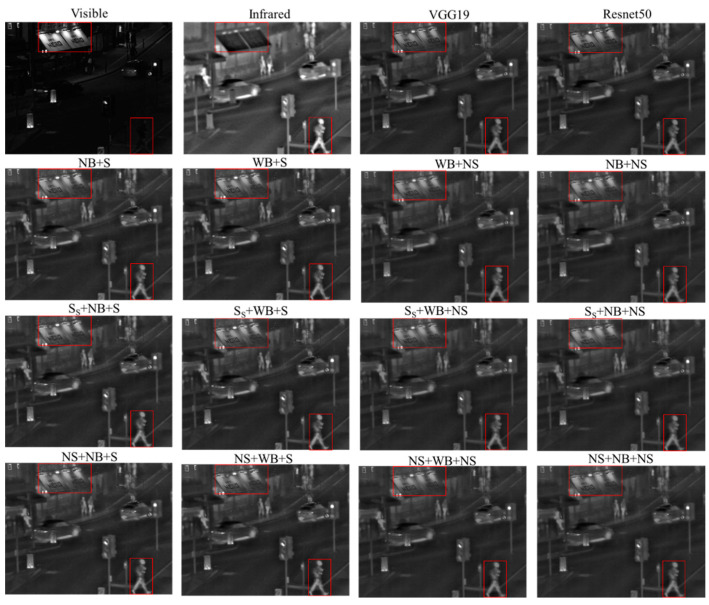
Fused images (NB, NS, NS_S_: Fused base part, fused salient part, and fused sparse part using Resnet50_ZCA_*l*_1_ norm, S, S_S_: Fused salient part and fused sparse part using summation, WB: Fused base part using weighted average). (The red box indicates the focal areas during the Subjective Assessments.).

The results of this section demonstrate the subjective evaluation of visual performance achieved by incorporating sparse components as part of the fusion process. In Figure 4, the outcomes of image fusion based on LatLRR are presented, showcasing the visual comparisons of different fusion strategies proposed in this study, including Resnet50_ZCA_l1 norm, weighted average, and summation. Despite the objective quantitative analysis indicating a significant enhancement in fusion performance with the inclusion of sparse parts, it is challenging for human observers to visually discern the differences in fusion outcomes when considering the limitations of human visual perception in the presence of sparse parts. We consider this to be an acceptable result, acknowledging that theoretically sparse parts contain less information, thus constraining human visual observation of these components. However, this does not imply their negligible importance, as evident from the quantitative results.

**Figure 5:**
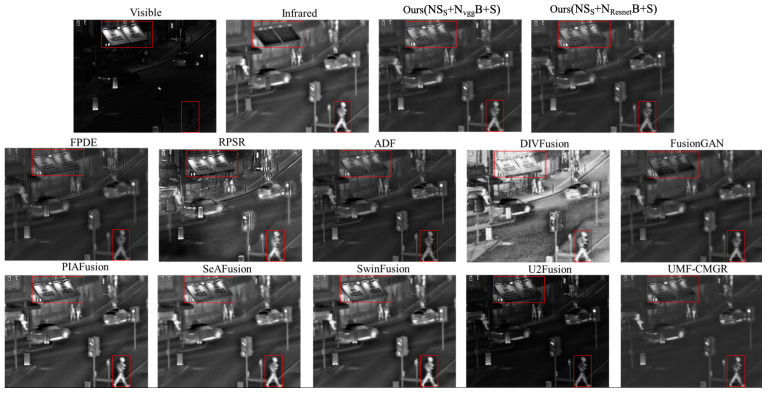
Comparison of fused images with SOTA methods. (The red box indicates the focal areas during the Subjective Assessments.).

Figure 5 illustrates the comparison between the proposed optimal fusion strategies (S_S_ + N_vgg_B + S, S_S_ + N_Resnet_B + S) and 10 other State-of-the-Art methods. The results demonstrate that the proposed method effectively showcases pedestrians and signage after the fusion of visible and infrared images, achieving satisfactory image fusion outcomes. This aligns with the quantitative evaluation results in Section 4.1, affirming the effectiveness and reliability of the proposed method. The other 10 methods also exhibit their unique fusion styles, as indicated by the quantitative results in Section 4.1, each having its own advantages. However, subjective assessments are susceptible to individual biases and inherent limitations in human visual perception. Therefore, the judgment of visual effects should be considered in the context of specific application scenarios.

As mentioned earlier in this study, previous image fusion methodologies based on LatLRR exclusively focused on fusion strategies concerning the base and salient parts, disregarding the sparse part by considering it as noise in the LatLRR image decomposition process. From the LatLRR theoretical perspective, these considerations are justifiable. Nevertheless, it is essential to note whether the sparse part solely comprises noise or potentially encapsulates crucial image information that could significantly contribute to improved fusion performance. The findings of this study demonstrate that regarding the sparse part as an innovative facet of the fusion process, unlike prior research, leads to substantial enhancements. The experimental validation of this concept in this study bears significant implications for LatLRR-based image fusion methodologies. Moreover, by amalgamating various fusion strategies and comparing their performance, this research proposes superior fusion strategies for consideration by subsequent researchers.

## 5. Discussion and Conclusions

In previous research, the image fusion strategies based on the LatLRR method predominantly focused on processing the base parts and salient parts, while regrettably disregarding the sparse parts. This oversight is noteworthy, as all three components originate from the original visible and infrared images. This operational approach risks the loss of crucial image information, failing to authentically represent the inherent characteristics of the original images, thus impeding the efficacy of the image fusion. Hence, this study advocates for the integration of the sparse parts as an integral element within the image fusion process. To articulate, it aims to concurrently amalgamate the base parts, salient parts, and sparse parts in strategies employed for visible and infrared image fusion.

Beyond employing conventional weighted average and summation techniques, this study leverages pre-trained CNN-based models, such as VGG19 and ResNet50, to extract image key features. It additionally integrates the *l*_1_ norm and ZCA+ *l*_1_ norm to derive weight maps. Findings from this study underscore that the inclusion of the sparse parts within the fusion process yields superior fusion performance compared to its exclusion. The incorporation of the sparse parts via CNN-based methodologies to obtain weight maps notably enhances the FMI_dct, FMI_w, and Nabf indicators. Amongst diverse strategy combinations, utilizing CNN-based methodologies to individually merge the base parts and sparse parts, while using summation to fuse the salient parts, emerges as the suggested fusion strategy within this study.

The fusion model amalgamating LatLRR with CNN effectively integrates the respective strengths of each method, thereby yielding a more robust and comprehensive fusion performance. The outcomes of this study bear substantial value for both academic and practical applications. They can be utilized in diverse multimodal sensing devices that incorporate visible and infrared imagery, such as drones, robots, or surveillance equipment, to enhance their sensing capabilities significantly.

## Figures and Tables

**Table 1:** Comparison of quantitative evaluation metrics (Resnet50_ZCA_*l*_1_ norm).

EN	MI	Qabf	FMI_Pixel	FMI_dct	FMI_w	Nabf	SCD	SSIM	MS_SSIM	Methods
6.2440	12.4881	0.3641	0.8985	0.3100	0.3487	0.0121	1.6506	0.7660	0.8676	NB + S
**6.2541**	**12.5082**	**0.3715**	0.8971	0.3467	0.3772	0.0163	**1.6528**	0.7671	0.8691	S_S_ + NB + S
6.2475	12.4951	0.3712	0.8981	0.3411	0.3784	0.0101	1.6520	0.7680	0.8687	NS_S_ + NB + S
6.1197	12.2395	0.2616	0.9071	0.3283	0.3531	0.0034	1.5979	0.7667	0.8300	WB + NS
6.1319	12.2637	0.2753	0.9040	0.3572	0.3742	0.0063	1.6004	0.7687	0.8319	S_S_ + WB + NS
6.1241	12.2481	0.2708	0.9063	0.3634	0.3845	0.0026	1.5995	0.7693	0.8315	NS_S_ + WB + NS
6.1394	12.2788	0.2652	0.9073	0.3290	0.3537	0.0034	1.6002	0.7666	0.8319	NB + NS
6.1512	12.3025	0.2788	0.9043	0.3578	0.3748	0.0063	1.6027	0.7686	0.8338	S_S_ + NB + NS
6.1433	12.2867	0.2740	0.9067	0.3646	0.3858	0.0026	1.6018	0.7692	0.8331	NS_S_ + NB + NS
6.2272	12.4543	0.3613	0.8982	0.3095	0.3484	0.0121	1.6483	0.7661	0.8659	WB + S
6.2373	12.4747	0.3686	0.8967	0.3462	0.3769	0.0162	1.6505	0.7671	0.8674	S_S_ + WB + S
6.2307	12.4614	0.3683	0.8978	0.3407	0.3781	0.0100	1.6497	0.7681	0.8670	NS_S_ + WB + S
6.1819	12.3639	0.3677	**0.9107**	0.4050	0.4168	0.0012	1.6348	0.7780	**0.8746**	VGG19
6.1953	12.3905	0.3510	0.9092	**0.4058**	**0.4169**	**0.0006**	1.6336	**0.7782**	0.8732	Resnet50

NB, NS, NS_S_: Fused base part, fused salient part, and fused sparse part using Resnet50_ZCA_*l*_1_ norm. S, S_S_: Fused salient part and fused sparse part using summation. WB: Fused base part using weighted average. Bold indicates the best-performing value for each indicator.

**Table 2:** Comparison of quantitative evaluation metrics (VGG19_*l*_1_ norm).

EN	MI	Qabf	FMI_Pixel	FMI_dct	FMI_w	Nabf	SCD	SSIM	MS_SSIM	Methods
6.2878	12.5755	0.3779	0.9013	0.3097	0.3478	0.0149	1.6728	0.7651	0.8709	NB + S
**6.2976**	**12.5952**	**0.3848**	0.8999	0.3465	0.3763	0.0193	**1.6750**	0.7661	0.8724	S_S_ + NB + S
6.2912	12.5824	0.3848	0.9008	0.3407	0.3772	0.0129	1.6742	0.7671	0.8720	NS_S_ + NB + S
6.1225	12.2449	0.2655	0.9073	0.3277	0.3531	0.0034	1.5999	0.7668	0.8311	WB + NS
6.1345	12.2691	0.2790	0.9043	0.3570	0.3745	0.0063	1.6023	0.7688	0.8330	S_S_ + WB + NS
6.1281	12.2561	0.2756	0.9068	0.3644	0.3862	0.0025	1.6020	0.7697	0.8333	NS_S_ + WB + NS
6.1875	12.3749	0.2871	0.9092	0.3269	0.3520	0.0059	1.6262	0.7659	0.8372	NB + NS
6.1988	12.3976	0.3000	0.9067	0.3568	0.3735	0.0089	1.6287	0.7679	0.8391	S_S_ + NB + NS
6.1914	12.3828	0.2959	0.9086	0.3622	0.3834	0.0051	1.6278	0.7686	0.8386	NS_S_ + NB + NS
6.2272	12.4543	0.3613	0.8982	0.3095	0.3484	0.0121	1.6483	0.7661	0.8659	WB + S
6.2373	12.4747	0.3686	0.8967	0.3462	0.3769	0.0162	1.6505	0.7671	0.8674	S_S_ + WB + S
6.2307	12.4614	0.3684	0.8978	0.3407	0.3781	0.0100	1.6497	0.7681	0.8670	NS_S_ + WB + S
6.1819	12.3639	0.3677	**0.9107**	0.4050	0.4168	0.0012	1.6348	0.7780	**0.8746**	VGG19
6.1953	12.3905	0.3510	0.9092	**0.4058**	**0.4169**	**0.0006**	1.6336	**0.7782**	0.8732	Resnet50

NB, NS, NS_S_: Fused base part, fused salient part, and fused sparse part using VGG19_*l*_1_ norm. S, S_S_: Fused salient part and fused sparse part using summation. WB: Fused base part using weighted average. Bold indicates the best-performing value for each indicator.

**Table 3:** Comparison of improvement after incorporating the sparse parts (Resnet50_ZCA_*l*_1_ norm).

EN	MI	Qabf	FMI_Pixel	FMI_dct	FMI_w	Nabf	SCD	SSIM	MS_SSIM	Methods
					(%)					
										NB + S
0.16	0.16	2.03	−0.16	11.83	8.19	−34.39	0.13	0.14	0.17	S_S_ + NB + S
0.06	0.06	1.96	−0.04	10.05	8.54	16.59	0.08	0.27	0.12	NS_S_ + NB + S
										WB + NS
0.20	0.20	5.23	−0.35	8.80	6.00	−86.37	0.16	0.26	0.23	S_S_ + WB + NS
0.07	0.07	3.49	−0.09	10.71	8.92	24.06	0.10	0.34	0.18	NS_S_ + WB + NS
										NB + NS
0.19	0.19	5.13	−0.33	8.76	5.97	−84.94	0.16	0.26	0.23	S_S_ + NB + NS
0.06	0.06	3.31	−0.07	10.85	9.08	24.44	0.10	0.34	0.15	NS_S_ + NB + NS
										WB + S
0.16	0.16	2.03	−0.16	11.84	8.20	−34.54	0.13	0.14	0.17	S_S_ + WB + S
0.06	0.06	1.95	−0.04	10.09	8.54	16.96	0.08	0.27	0.12	NS_S_ + WB + S

ID_S: The metrics derived from methods incorporating the sparse parts. ID_non: The metrics derived from methods that do not involve the sparse parts. Improvement: (ID_S − ID_non)/ID_non × 100. Nabf values should be multiplied by −1 because a smaller value indicates better performance.

**Table 4:** Comparison of improvement after incorporating the sparse parts (VGG19_*l*_1_ norm).

EN	MI	Qabf	FMI_Pixel	FMI_dct	FMI_w	Nabf	SCD	SSIM	MS_SSIM	Methods
					(%)					
										NB + S
0.16	0.16	1.81	−0.15	11.87	8.21	−29.23	0.13	0.14	0.17	S_S_ + NB + S
0.05	0.05	1.83	−0.05	10.00	8.46	13.19	0.08	0.26	0.12	NS_S_ + NB + S
										WB + NS
0.20	0.20	5.10	−0.33	8.93	6.04	−85.40	0.15	0.26	0.23	S_S_ + WB + NS
0.09	0.09	3.83	−0.06	11.19	9.35	27.87	0.13	0.38	0.27	NS_S_ + WB + NS
										NB + NS
0.18	0.18	4.49	−0.27	9.15	6.11	−51.31	0.15	0.26	0.23	S_S_ + NB + NS
0.06	0.06	3.08	−0.06	10.80	8.93	14.21	0.09	0.34	0.17	NS_S_ + NB + NS
										WB + S
0.16	0.16	2.03	−0.16	11.84	8.20	−34.54	0.13	0.14	0.17	S_S_ + WB + S
0.06	0.06	1.95	−0.04	10.08	8.55	17.01	0.09	0.27	0.12	NS_S_ + WB + S

ID_S: The metrics derived from methods incorporating the sparse parts. ID_non: The metrics derived from methods that do not involve the sparse parts. Improvement: (ID_S − ID_non)/ID_non × 100. Nabf values should be multiplied by −1 because a smaller value indicates better performance.

**Table 5:** Comparison of quantitative evaluation metrics with SOTA methods.

EN	MI	Qabf	FMI_Pixel	FMI_dct	FMI_w	Nabf	SCD	SSIM	MS_SSIM	Methods
6.2519	12.5037	0.3870	0.8827	0.2256	0.2519	0.1460	1.6147	0.7070	0.8648	FPDE [14]
7.1105	14.2209	0.3848	0.8826	0.1928	0.2569	0.2448	1.3986	0.6603	0.8458	RPSR [21]
6.2691	12.5382	0.4127	0.8829	0.2275	0.2595	0.1451	1.6133	0.7091	**0.8760**	ADF [15]
**7.5980**	**15.1960**	0.2831	0.8567	0.1996	0.2399	0.4225	1.4331	0.5429	0.7264	DIVFusion [46]
6.3946	12.7893	0.1852	0.8863	0.1702	0.1933	0.0937	1.3831	0.6279	0.7009	FusionGAN [40]
6.7471	13.4943	**0.4273**	**0.9049**	0.2163	0.2628	0.2138	1.5967	0.6701	0.8233	PIAFusion [27]
7.0462	14.0925	0.4014	0.8958	0.2030	0.2511	0.3072	1.6298	0.6457	0.8251	SeAFusion [26]
6.7612	13.5225	0.4059	0.9014	0.2100	0.2544	0.1847	1.6307	0.6865	0.8292	SwinFusion [53]
6.8810	13.7621	0.3667	0.8869	0.2137	0.2468	0.3336	**1.7128**	0.6454	0.8729	U2Fusion [29,62]
6.5667	13.1333	0.3291	0.8898	0.2031	0.2331	0.1859	1.6164	0.6865	0.8437	UMF-CMGR [28]
6.2912	12.5824	0.3848	0.9008	0.3407	0.3772	0.0129	1.6742	0.7671	0.8720	Ours(NS_S_ + N_vgg_B + S)
6.2475	12.4951	0.3712	0.8981	**0.3411**	**0.3784**	**0.0101**	1.6520	**0.7680**	0.8687	Ours(NS_S_ + N_Resnet_B + S)

N_vgg_B, N_Resnet_B: Fused base part using VGG19_*l*_1_ norm and Resnet50_ZCA_*l*_1_ norm, respectively. S, S_S_: Fused salient part and fused sparse part using summation. Bold indicates the best-performing value for each indicator.

## Data Availability

Publicly available datasets were analyzed in this study. These data can be found here: [https://doi.org/10.6084/m9.figshare.1008029.v2], accessed on 20 August 2023.
